# Perspectives on Recent Progress in Focused Ultrasound Immunotherapy

**DOI:** 10.7150/thno.37131

**Published:** 2019-10-15

**Authors:** Natasha D. Sheybani, Richard J. Price

**Affiliations:** Department of Biomedical Engineering, University of Virginia, Charlottesville, VA

**Keywords:** focused ultrasound, immunotherapy, cancer, targeted drug and gene delivery

## Abstract

Immunotherapy holds tremendous promise as a strategy for eradicating solid tumors. However, poor T cell infiltration and persistence within most solid tumor microenvironments, as well as mechanisms of adaptive resistance, continue to severely limit the accessibility of most immunotherapies to a broad patient population. This limitation perpetuates the demand for allied therapeutic strategies. Among such strategies is focused ultrasound (FUS), a non-invasive, non-ionizing technique for precisely targeted acoustic energy deposition into tissues. FUS has gained remarkable attention over recent years as a modality for elicitation of immune mechanisms in cancer and other pathologies. In 2017, we published a comprehensive review paper detailing existing evidence for immune modulation and therapy with FUS, as well as impending challenges and opportunities of consideration for the field. Over the last two years, a multitude of clinical trials have come online to explore safety, feasibility, and efficacy of FUS for cancers of the brain and periphery - including the first clinical trial to combine FUS with immunotherapy. Moreover, the last two years have seen a surge in FUS immunotherapy presentations at therapeutic ultrasound scientific meetings. Given the burst of activity in this field, we submit that an update on FUS immunotherapy progress is timely. In this review, we offer an updated overview and perspectives on scientific and clinical development in the FUS immunotherapy domain.

## Introduction

Immunotherapies are treatments designed to mobilize endogenous immune mechanisms to effectively mount an attack against cancerous cells, all the while preserving the integrity of normal or healthy cells. Because immunity plays such a pivotal role in tumor evolution, it holds that immunotherapies have tremendous promise as strategies for tumor eradication. Recent decades have seen substantial advancement in immunotherapy interventions capable of eliciting unparalleled therapeutic outcomes; such strategies include checkpoint blockade (e.g. anti-PD1) 1-3, peptide-based vaccination 4, and adoptive transfer of genetically modified T cells targeting tumor-specific antigens 5. Despite this promise, only a small proportion of patients (15-40% dependent on cancer type) with solid tumors realize these benefits at present, owing to poor T cell infiltration and persistence within the tumor microenvironment (TME) 6. This limitation perpetuates the demand for therapeutic platforms that boost the immunogenicity of tumors while curbing the onset of adaptive resistance mechanisms, effectively converting immunologically “cold” (poorly infiltrated) tumors into immunologically “hot” (well-infiltrated) ones 7. Adjuvant strategies - such as dual checkpoint blockade, ionizing radiation, and CpG injections - while postulated to sensitize the TME to immunotherapy, are inherently limited by severe off-target autoimmune-related toxicities 8,9. Focused ultrasound (FUS) - a safe, repeatable, non-invasive and *non-ionizing* technique for localized acoustic energy deposition - is yet another distinct approach that has demonstrated promise for eliciting immune responses that may synergize with immunotherapy 10.

In 2017, we (NDS and RJP) served as co-authors on a comprehensive review article in *Theranostics* that covered the novelty and significance of FUS for immunotherapy as well as the state of the field, with a focus on challenges and opportunities in the central nervous system (CNS) 11. We offered an overview of physical mechanisms of FUS; antibody, cytokine and cell delivery to the brain; growing pre-clinical and clinical evidence of FUS immunomodulation within and outside the CNS; and perspectives to inform the future of this emerging topic. Over the last two years, the FUS community has seen a steady and remarkable growth in immunotherapy activity. Since 2017, the first-in-human clinical trial combining FUS with immunotherapy has come online, as well as a multitude of others that are exploring safety, feasibility, and efficacy of FUS for cancer therapy within and outside the CNS. These trials are imperative to the establishment of a foundation for eventual interrogation of immunomodulation and immune therapy combinations with FUS.

Since the time of our last review, a handful of findings have emerged in the literature that will be discussed herein. However, it is worth emphasizing what is not immediately evident from the literature; while many groups are working in this space presently, publications from this new wave of research are just now beginning to emerge. FUS immunotherapy has gained notable traction as evidenced by the number of oral and poster presentations at international meetings including the 6^th^ International Symposium on Focused Ultrasound (October 2018) and the most recent International Society for Therapeutic Ultrasound Annual Symposia (May 2018; June 2019). Despite these meetings being relatively small, ~50 abstracts on FUS immunotherapy were presented. In this brief review, we will offer updated perspectives on progress within the field, with an emphasis on studies performed since our 2017 review in *Theranostics*.

## Physical Mechanisms of Focused Ultrasound

FUS is a non-invasive, non-ionizing technique for high-density acoustic energy deposition. It has gained legitimacy and momentum as an approach for targeted tumor disruption via thermal and/or mechanical mechanisms, which are comprehensively detailed in other reviews to which interested readers are herein referred 12,13. Typically performed under ultrasound or MR image guidance, FUS offers a versatile range of focal bioeffects that are broadly contingent upon the frequency, power, duration, and duty cycle of sonication. In general, FUS-elicited bioeffects are typically considered as being either predominantly “thermal” or “mechanical” in nature. In essence, thermal FUS regimens generate focal temperatures exceeding 60^o^C, leading to nearly instantaneous onset of coagulative necrosis in the focal zone and thermal stresses in the periablative zone 14,15. At lower intensities, these regimens can give rise to subablative heating, i.e. hyperthermia, which is characterized by the predominance of non-lethal heat stress signatures. Meanwhile, mechanical FUS leads to acoustic cavitation, acoustic streaming/microstreaming, radiation force, and shear stresses within the pulsed acoustic field 16. At high intensities, these bioeffects cause mechanical lysis of cells with minimal temperature rise; mechanical lesioning often results in subcellular fragmentation with sharply delineated margins 16. In the presence of systemically circulating microbubbles (MB), the intensity threshold required to achieve these cavitation effects within the tissue is lowered. Low intensity FUS with circulating MB is a form of mechanical FUS energy deposition that has been shown to elicit transient opening of tight junctions, sonoporation of vascular endothelium, and enhanced capacity for transcytosis; these effects have been exploited for blood brain barrier (BBB) and/or blood-tumor barrier (BTB) disruption 17. This disruption can facilitate delivery of various agents to the brain, including chemotherapies, drug and gene-bearing polymeric nanoparticles, and antibodies 18-26.

**Figure 1:**
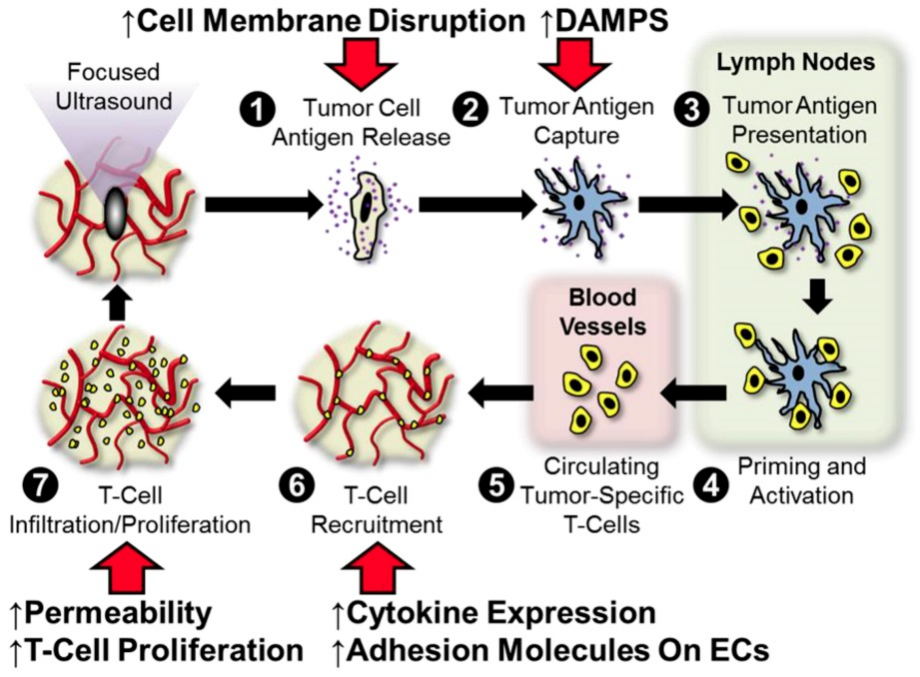
** Hypothesized points of intersection between focused ultrasound and the cancer immunity cycle**. In the cancer immunity cycle, antigens (purple) released from tumor cells (tan; 1) are captured by dendritic cells (blue; 2) and presented to T-cells (yellow; 3) in lymph nodes (light green), leading to priming and activation of effector T-cells (4). Activated effector T-cells then pass into the systemic circulation (light pink; 5) and are trafficked to the tumor via adhesion to tumor endothelium (6). T-cells recruited from the circulation then infiltrate the tumor (7), where they specifically recognize and subsequently kill tumor cells. Tumor cell killing serves to release more antigen (1), allowing the cycle to continue. We hypothesize that focused ultrasound can trigger and/or boost the anti-cancer immunity by intersecting at several points (red arrows) in this cycle. These include (i) enhanced tumor antigen release by cell membrane disruption, (ii) improved dendritic cell maturation via enhanced expression of damage-associated molecular patterns (DAMPS, i.e. alarmins), (iii) greater antigen flow to lymph nodes and less restricted intra-tumor T-cell migration as a result of mechanical disruption of stroma, and (iv) altered cytokine production, which may lead to augmented endothelial adhesion molecule expression and/or proliferation of intra-tumor T-cells. Adapted from Curley et al. (2017).

The concept of FUS immunotherapy finds its roots in the supposition that these regimens are distinctly capable of stimulating anti-tumor immune mechanisms that include tumor-specific inflammation, tumor-associated antigen and alarmin liberation, cytokine modulation, leukocyte infiltration and activation, and/or curbing of immunological tolerance. Figure 1 outlines points at which we hypothesize that FUS may interface with the traditional cancer-immunity cycle 11. Briefly, we hypothesize that FUS exposure is capable of (i) liberating, if not altering the repertoire of, tumor antigens following cell membrane disruption, (ii) improving dendritic cell maturation via enhanced expression of DAMPS (alarmins), (iii) elevating antigen flow to lymph nodes and alleviating barriers to intratumoral T-cell migration as a result of mechanical disruption of stroma, and (iv) altering cytokine production, which may lead to augmentation of endothelial adhesion molecule expression and/or proliferation of effectors within the tumor. We anticipate that these hypothesized impacts of acoustic exposure on the tumor-immune landscape will be differentially elicited by ablative and non-ablative FUS regimens. A more detailed discussion of these postulated intersections between FUS and the cancer immunity cycle is provided in our 2017 review. Below, we review several pre-clinical studies since 2017 that have lent further insight into these hypotheses, as well as ongoing clinical studies that are setting the stage for future investigation.

## New Clinical Trials: Setting the Stage for the Translation of FUS Immunotherapies

Transcranial FUS has been explored as an intervention for a variety of neurological pathologies including neurodegenerative diseases, primary and metastatic brain tumors, neuropathic pain, and psychiatric disorders 27. However, to date, no preclinical or clinical investigations have combined FUS with immunotherapy for malignancies of the CNS. The highly exclusionary nature of the BBB - as well as the relative immunological silence of normal brain (low MHC expression and lymphocyte traffic) - remain challenges to effective CNS immunotherapy. However, the future outlook is promising for such combinatorial paradigms in the brain owing to important recent developments. The last two years have seen a significant surge in clinical activity exploring brain applications of FUS. The key outcomes of clinical studies exploring FUS BBB opening are summarized in Table 1. We highlight below some of the clinical milestones that have demonstrated the safety and feasibility of FUS for CNS pathologies.

**Figure 2:**
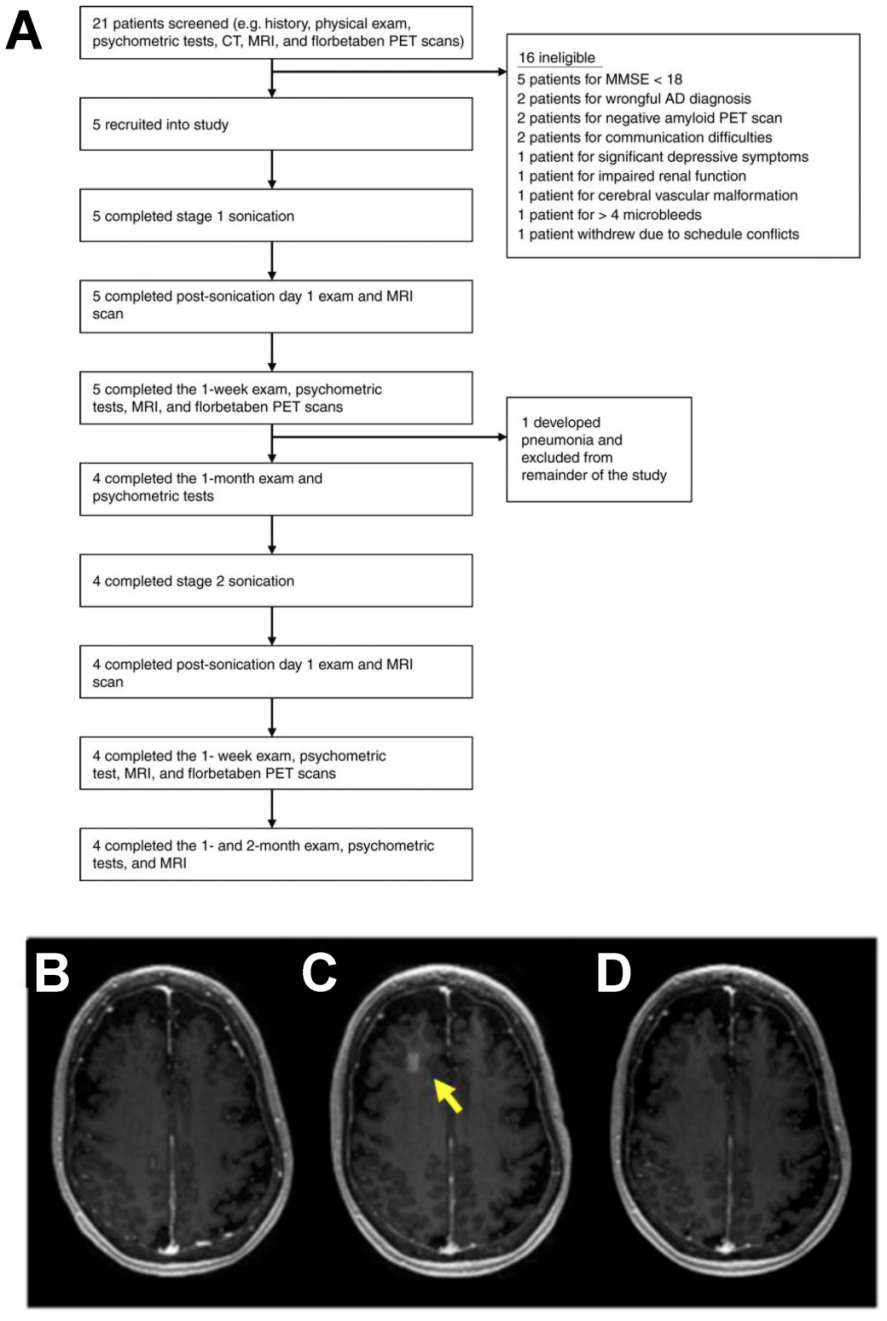
** Clinical trial for blood-brain barrier opening in Alzheimer's disease patients.** A. Overview of the study. Flow chart illustrates the study design and overview of patients screened and enrolled in the study. B-D. MRI demonstration of blood-brain barrier opening and closure. Axial T1-weighted gadolinium MR images of a patient at baseline (B), immediately after stage 2 sonication and blood-brain barrier (BBB) opening (C), and at 24 h after procedure (D). Contrast extravasation within the 10 × 10 × 7 mm3 sonicated volume in the right frontal lobe is seen immediately after the procedure, demonstrating increased BBB permeability. At 24 h after the procedure, there was no significant extravasation of contrast in the area, suggesting BBB closure. Adapted from Lipsman et al. (2018).

Ultrasound treatment has been demonstrated to confer clearance of amyloid plaques in transgenic models of Alzheimer's disease, independent of drug or gene delivery 28,29. These results implicated a putative mechanism based on an immune response to ultrasound, as sonicated regions were enriched for markers of microglial activation and greater localization of amyloid beta was observed within microglia. These findings motivated the evaluation of FUS BBB disruption for patients with early to moderate Alzheimer's disease. In 2018, the first clinical findings on safety and feasibility of BBB disruption using i.v. MB and the ExAblate MRI-guided transcranial FUS system (Insightec) were reported in Alzheimer's patients (Figure 2) 30. While the study did not elucidate any clear effect of FUS BBB disruption on beta-amyloid deposition, it is important to note that this trial was not designed to study efficacy. Rather, this study generated support for reversibility, repeatability, and safety of FUS BBB opening. Additionally, low-intensity FUS BBB opening with systemically administered chemotherapy (i.e. liposomal doxorubicin or temozolomide) was reported as safe and feasible in patients with high-grade glioma 31. Patients underwent the procedure one day prior to surgical resection, wherein tissue specimens of FUS-exposed and non-exposed regions were collected for further analysis. Due to low sample sizes and complications with sample integrity, the group was unable to draw firm conclusions about the influence of FUS-mediated BBB opening on drug delivery. In contrast to the MRI-guided approach, the SonoCould-1 (CarThera) achieves transient BBB disruption via low-intensity pulsed ultrasound using an implantable device 32. In a recent clinical trial, the SonoCloud-1 was employed for monthly transient BBB disruption prior to i.v. carboplatin administration in recurrent glioblastoma patients. The trial demonstrated the safety, feasibility and tolerability of large volumetric BBB opening with an implantable device 33. Moreover, it was observed that patients who received the treatment benefited from extended progression-free and overall survival. The authors suggest that this was consistent with their observations that tumor growth may have been more effectively controlled within sonication regions. Since the focal volume of the device was unable to cover the entirety of the tumor, future efforts will be aimed at enabling a larger treatment envelope to improve efficacy of the approach.

These studies come at a time when perspectives framing the CNS as a site of “immune privilege” are rapidly evolving into ones that instead consider the CNS to be “immunologically distinct.” With the continued emergence of discoveries that connect the CNS and immune system, the tractability of leveraging FUS for immunotherapy in the brain is clearly growing.

Since the benefits of immunotherapy have been realized more extensively for extracranial tumors, new and ongoing clinical trials utilizing FUS in the treatment of neoplasms outside of the CNS will also be vastly informative to the development of FUS immunotherapy. Beginning in 2014, a clinical trial at the University of Virginia investigated the safety and feasibility of thermally ablating benign breast fibroadenomas using the CE-marked Theraclion Echopulse system (ultrasound-guided FUS device) (NCT02078011) 34. This trial offered invaluable insight into the execution of an ultrasound-guided FUS workflow for ablation of breast lesions. Following on the success of this initial trial, a larger multi-center trial is currently under accrual for evaluation of FUS safety and efficacy in breast fibroadenoma patients (NCT03044054). More importantly, however, the breast fibroadenoma trial set a precedent for exploration of FUS thermal ablation in the setting of breast malignancies.

**Figure 3:**
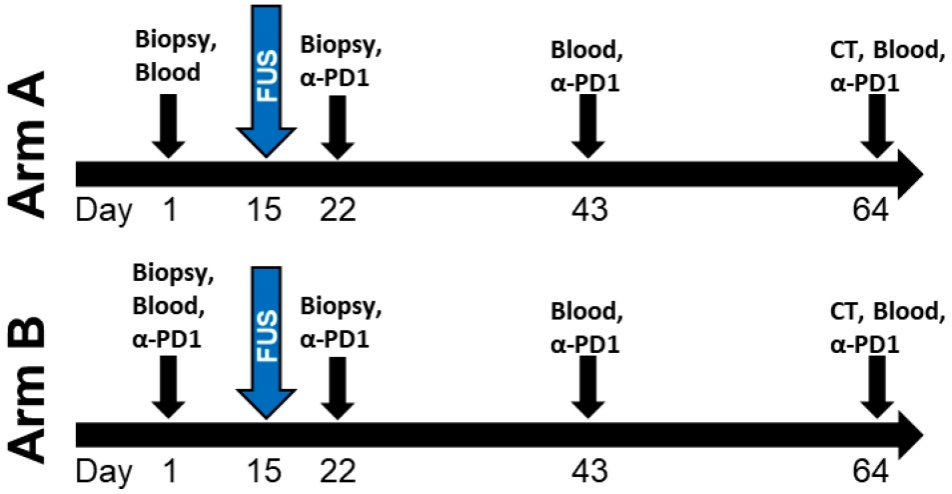
** Design of University of Virginia clinical trial (NCT03237572) combining thermally ablative FUS with pembrolizumab (α-PD1) for metastatic breast cancer.** Patients on Arm A of the trial first receive pembrolizumab 1 week after FUS ablation of the primary tumor, while those on Arm B first receive pembrolizumab 2 weeks before the FUS procedure.

**Figure 4:**
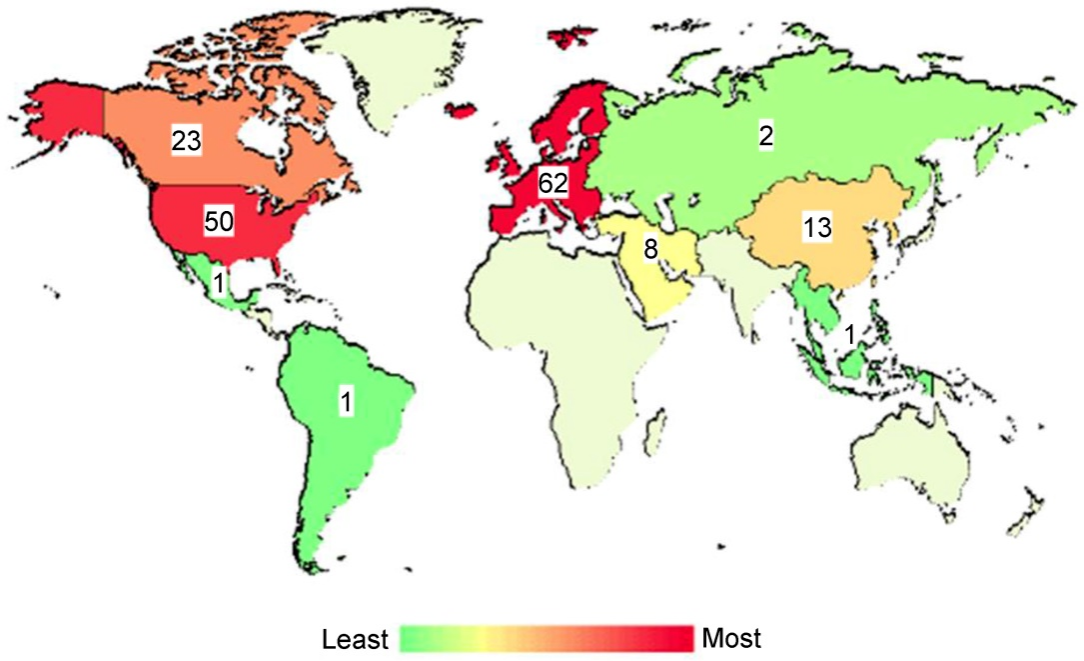
** Global distribution of clinical trials evaluating focused ultrasound for cancer applications.** Across the globe, there are a number of clinical trials investigating the safety, feasibility or efficacy of focused ultrasound for treatment of primary or disseminated solid tumors. Studies depicted herein represent those that are either not yet recruiting, recruiting, or completed. Color intensities on the map correspond with number of trials with a location in that region. Numerical labels refer to the exact number of clinical trials. Adapted from ClinicalTrials.gov.

Owing to this previously established use of Theraclion's Echopulse system at the University of Virginia, a FUS immunotherapy milestone was achieved in 2017 with the initiation of a clinical trial evaluating the combination of FUS thermal ablation with pembrolizumab in metastatic breast cancer patients (NCT03237572). Although the trial has not yet fully accrued, several women have been treated thus far. The design of the trial is shown in Figure 3. Aside from this trial, there are over 150 other listed, recruiting, or completed clinical trials worldwide exploring FUS for cancer treatment. The worldwide distribution of these trials is illustrated in Figure 4. The diversity of these trials with respect to cancer type, FUS technology, exposure conditions, and patient backgrounds presents a rich opportunity for data mining to determine how best to direct future immunotherapy trials with respect to these factors. As the outcomes of these clinical trials are published, they will have the potential to inform future efforts seeking to combine FUS with immunoadjuvants.

## New Pre-Clinical Investigations into How Mechanical FUS Parameters Influence Inflammation

A handful of recent studies have probed relationships between various mechanical FUS parameters for BBB opening and the elicitation of local inflammatory responses. As the field of FUS-immunotherapy advances into the treatment of CNS neoplasms, these studies will likely provide valuable insight into how mechanical FUS may be tuned to possibly synergize with immunotherapies.

In 2016, it was reported that FUS-mediated BBB opening is capable of eliciting sterile inflammation in naïve brain 36. In longer term follow-up studies, six weekly pulsed FUS and MB treatments led to long-term signatures consistent with pathological injury to the neurovascular unit, in contrast with single sonication; specifically, multiple sonications elicited hypointense signatures on T2* MRI (suggestive of RBC extravasation due to vascular damage), cortical atrophy, and astrogliosis. Moreover, histological studies revealed that multiple FUS exposures resulted in increased activated microglia, infiltrating CD68+ macrophages, and hyperphosphorylated Tau (pTau) and neurofibrillary tangle elevation in neurons in the sonicated region of the brain 37. Such signatures as increased presence of pTau and microglial activation are consistent with the chronic inflammatory component of neurodegenerative processes such as those present in Alzheimer's disease.

During and after the publication of these sterile inflammation studies, other investigators published studies emphasizing how these effects are dependent on sonication parameters (e.g. acoustic pressure), as well as other parameters such as MB formulation, choice of anesthesia, carrier gas 38, and MB infusion rate 37,39-41. The importance of acoustic feedback in controlling BBB opening regimens for fine tuning of parameters that determine acute inflammatory responses has also been highlighted 39.

In particular, MB dose is known to be a critical parameter for instigation of neuroinflammatory effects in the brain. In a study comparing the recommended MB dose for clinical imaging with a dose 10x greater, FUS BBB permeabilization of rat dorsal hippocampal microvessels with the higher MB dose acutely induced elevated transcription of proinflammatory cytokine genes (i.e. Ccl2, Ccl3, Ccl7, Cxcl1, Cxcl11, Il1b, and Il6) - all of which was mostly resolved by 24 hrs. EC activation and astrocyte activation were noted via Sele and Gfap gene expression, respectively. Interestingly, there was no elevation in gene expression for endothelial adhesion molecules Vcam1 or Icam1 - ligands key to leukocyte extravasation out of the vasculature - at the time points evaluated 42. Nonetheless, upregulation of other key immune activation genes including Tnf, Birc3, and Ccl2 drew attention to MB dose as a significant modulator of acute inflammation following FUS BBB disruption. Higher MB dose was further determined to stimulate NFkB signaling, microhemorrhage, edema, neuronal damage, and neutrophil infiltration 40. Thus, appropriate MB dose selection will be critical to the invocation and tuning of inflammatory responses to FUS in future clinical studies.

Taken together, these studies provide evidence that certain signatures of immune activation can occur in the brain with FUS. Factors such as sonication parameters, MB dose, anesthesia, model, MB formulation, and timing of onboard therapies relative to treatment are thus important considerations for those conducting FUS immunotherapy studies. Despite the promise of FUS to lift barriers to effective cancer immunotherapy applications in the brain, there remains an unresolved contention between maintaining safety to healthy tissue and causing immunogenic “damage” to malignant tissue. It is possible that with appropriately tuned exposure conditions, acoustic energy can be leveraged to better enable immunotherapies not only via improved delivery, but also via favorable modes of immune modulation. It is of note, however, that the capacity of FUS to elicit adaptive resistance mechanisms or modulate immunosuppressive cell populations remains poorly understood across tumor types 43.

## New Pre-Clinical Investigations Combining FUS with Checkpoint Inhibitors

Beyond the brain, a handful of notable strides have been made to advance the combination of FUS with immunotherapies in the periphery. For example, the combination of toll-like receptor agonist CpG, anti-PD1 and FUS thermal ablation in murine B16 melanoma was shown to dramatically increase local and systemic immunity compared to monotherapies 44. Combining FUS with CpG elicited substantial skewing of gene expression toward markers like F4/80, Cd11b, and Tnf in the ablated tumor, while distant tumors upregulated Cd11c, Cd3, and Ifng. Ablation alone enhanced antigen cross-presentation intratumorally but not systemically, suggesting that the effect of ablation independent of priming via additional immune adjuvants was insufficient to mount a robust memory response. Amplification of systemic antigen cross-presentation, type 1 interferon release, and CD169+ myeloid cell recruitment were observed in the setting of ablation combined with immunotherapy - suggestive that this paradigm was capable of enriching for a unique class of antigen-presenting cells that cross-prime independent of DCs. In an adaptation of this combinatorial approach in murine breast cancer, the combination of single dose thermally activatable doxorubicin-loaded liposomes with FUS hyperthermia and an adjuvant agonistic immunotherapy priming protocol (anti-PD1 + CpG) elicited treated and distant tumor eradication, as well as significant survival benefit 45. In this study, complete response rate was greatest in mice when immunotherapy priming was conducted prior to administering a single dose of chemotherapy.

Sonodynamic therapy (SDT), an emerging technique for exerting tumor cell death through combination of low intensity ultrasound with chemical sonosensitizers, is under investigation as a strategy for cancer vaccination. A combinatorial therapeutic paradigm employing SDT with sonosensitizers/imiquimod-loaded liposomes and anti-PD-L1 has recently been demonstrated to exert efficacy against 4T1 mammary and CT26 colorectal carcinomas 46. This allied strategy - wherein anti-PD-L1 therapy was initiated after SDT - restricted primary tumor outgrowth, prevented lung metastases and conferred augmented immunological memory function that protected mice upon tumor re-challenge. Separately, HiPorfin-induced SDT has been demonstrated to promote the expression of calreticulin - a key marker of immunogenic cell death - on the surface of Hep3b human liver cancer cells. This regimen was also effective in generating an abscopal effect and immunological memory against murine H22 tumors 47.

Lastly, a couple of recent investigations into the immunological impact of mechanical acoustic energy deposition have emerged over the last two years. Boiling hisotripsy (BH) has been demonstrated to promote transient elevations of plasma and intrarenal TNF, high-mobility group box 1 (HMGB1), IL-10, and IL-6 in a rat model of renal cell carcinoma 48. These trends consistent with an acute inflammatory response were accompanied by an elevation in CD8+ T cells across both BH ablated and untreated contralateral tumors at 48 hours. Moreover, “anti-vascular” ultrasound treatment with MB has recently been combined with anti-PD1 therapy 49. While the combination conferred tumor growth constriction and enhanced survival resulting from combinatorial therapy in a CT26 model of murine colon carcinoma, anti-tumor responses were not conclusively linked to a T cell-dependent mechanism.

**Figure 5:**
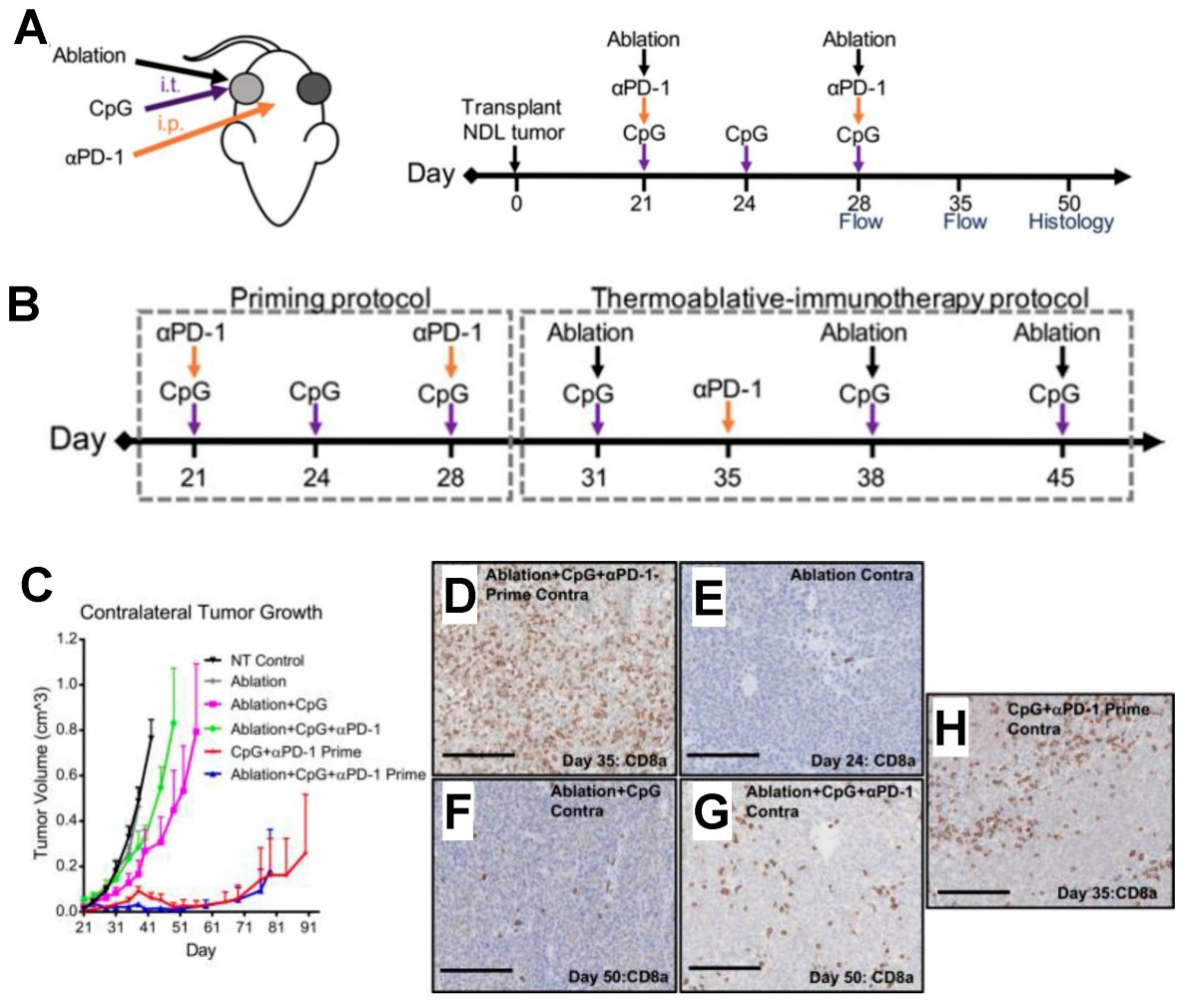
** Incorporation of priming into FUS thermal ablation immunotherapy protocols.** A. Regimen of coincident thermally ablative (TA) immunotherapy, intratumoral CpG and i.p. anti-PD-1 (αPD-1) in mice orthotopically transplanted with NDL tumor biopsies in the fourth and ninth mammary fat pad. B. Regimen of primed TA immunotherapy. Immunotherapy was administered prior to thermal ablation in priming protocol. Following priming, mice received a combination of thermal dosing and immunotherapy in TA-immunotherapy protocol. Treatments included CpG + αPD-1-Prime, ablation + CpG + αPD-1-Prime (Abl + CpG + αPD-1-Prime), and NT control. CpG was injected and αPD-1 was injected i.p. C. Priming prior to thermal ablation (ablation + CpG + αPD-1-Prime) suppressed contralateral tumor growth compared with other treatments that incorporated ablation, including NT control, ablation, ablation + CpG, and ablation + CpG + αPD-1. For bilateral tumors, the growth of primed TA-immunotherapy was similar to primed immunotherapy alone. Data are plotted as mean ± SEM. (D-H) Infiltrating CD8+ T cells (brown stain) in contralateral tumors were increased by (D) primed TA-immunotherapy as compared with (E) ablation, (F) ablation + CpG, (G) coincident TA-immunotherapy, and (H) primed. Scale bars: 150 μm. Adapted from Silvestrini et al. (2017).

The above highlighted studies represent some of the earliest published efforts to combine FUS with immunotherapies such as checkpoint blockade across a diverse array of physical regimens. Publications emerging in the field have called immunological priming to attention as a means towards achieving effective FUS immunotherapy. Notably, these studies arrived at priming as an important consideration when coincident immunotherapy protocols did not yield similar success 43-45 (Figure 5). However, other studies have seen efficacy in the context of checkpoint inhibition similarly targeting the PD-1/PD-L1 axis without the need for priming 46. This suggests that, despite the ability of priming to facilitate FUS immunotherapy, further testing of approaches that involve coincident or delayed immunotherapy relative to FUS without priming are warranted - especially since the importance of immunotherapy timing has yet to be established across FUS regimens, immunological adjuvants, and tumor models.

## Conclusions & Outlook

To date, no studies have evidenced the capacity of FUS monotherapy to achieve immunological tumor control independent of adjuvant therapy. Through appropriate tuning of exposure conditions and comprehensive immunological characterization, the prospect of unmasking the utility of FUS as an immunomodulatory monotherapy may be attainable. Since FUS immunotherapy is in its earliest stages, it is paramount to the field that such formative variables as immunotherapy timing, FUS exposure conditions, choice of immunoadjuvant, and differential response across tumor models be resolved over the coming years. We are optimistic that with the continuation of pre-clinical development and clinical translation at the current pace, FUS will surface as a transformative modality for combination with immunotherapies.

## Figures and Tables

**Table 1:** Summary of FUS BBB opening clinical trials completed to date.

Clinical Trial Identifier	Location	Condition	Device	IV Contrast Agent	Outcomes	Ref.
NCT02986932	Sunnybrook Health Sciences Centre (Canada)	Early stage Alzheimer's Disease	ExAblate Transcranial (220 kHz) system	Definity	• Non-invasive MRgFUS BBB opening is safe, reversible, and repeatable in amyloid-positive AD. • No group-wise change in amyloid levels were detected post-sonication. • No clinically significant departures from baseline were noted in patient cognition or daily functioning.	35
NCT02253212	Groupe Hospitalier Pitié Salpetriere (France)	Recurrent glioblastoma	SonoCloud	SonoVue	• Repeat BBB opening using an implantable pulsed ultrasound device with systemic MB injection is safe and well tolerated in recurrent glioblastoma patients. • Evidence of restricted tumor progression in sonication zone and its surroundings on MRI suggesting that treatments may increase effectiveness of systemic drugs such as carboplatin without inducing neurotoxicity.	32,33
NCT02343991	Sunnybrook Health Sciences Centre (Canada)	Primary brain tumor/Glioma	ExAblate Transcranial (220 kHz) system	Definity	• Transient MRgFUS BBB opening is well-tolerated, safe, reversible, and feasible in tumor and peritumor tissue. • Systemic chemotherapy delivery with BBB opening is safe and feasible.	31
NCT03626896	Linkou Chang Gung Memorial Hospital (Taiwan)	Recurrent glioblastoma	NaviFUS system	SonoVue	Trial completed. Results forthcoming.	N/A

## References

[1] Simeone E, Ascierto PA (2012). Immunomodulating antibodies in the treatment of metastatic melanoma: the experience with anti-CTLA-4, anti-CD137, and anti-PD1. J Immunotoxicol.

[2] Liu J, Yuan Y, Chen W, Putra J, Suriawinata AA, Schenk AD, Miller HE, Guleria I, Barth RJ, Huang YH, Wang L (2015). Immune-checkpoint proteins VISTA and PD-1 nonredundantly regulate murine T-cell responses. Proc Natl Acad Sci U S A.

[3] Curran MA, Montalvo W, Yagita H, Allison JP (2010). PD-1 and CTLA-4 combination blockade expands infiltrating T cells and reduces regulatory T and myeloid cells within B16 melanoma tumors. Proc Natl Acad Sci U S A.

[4] Parmiani G, Russo V, Maccalli C, Parolini D, Rizzo N, Maio M (2014). Peptide-based vaccines for cancer therapy. Hum Vaccin Immunother.

[5] Peng W, Liu C, Xu C, Lou Y, Chen J, Yang Y, Yagita H, Overwijk WW, Lizee G, Radvanyi L, Hwu P (2012). PD-1 blockade enhances T-cell migration to tumors by elevating IFN-gamma inducible chemokines. Cancer Res.

[6] Onyshchenko M (2018). The Puzzle of Predicting Response to Immune Checkpoint Blockade. EBioMedicine.

[7] Erdag G, Schaefer JT, Smolkin ME, Deacon DH, Shea M, Dengel LT, Patterson JW, Slingluff CL (2012). Immunotype and Immunohistologic Characteristics of Tumor-In fi ltrating Immune Cells Are Associated with Clinical Outcome in Metastatic Melanoma. Cancer Res.

[8] Kroeze SGC, Fritz C, Hoyer M, Lo SS, Ricardi U, Sahgal A, Stahel R, Stupp R, Guckenberger M (2017). Toxicity of concurrent stereotactic radiotherapy and targeted therapy or immunotherapy: A systematic review. Cancer Treat Rev.

[9] Gelao L, Criscitiello C, Esposito A, Goldhirsch A, Curigliano G Immune Checkpoint Blockade in Cancer Treatment: A Double-Edged Sword Cross-Targeting the Host as an “Innocent Bystander.” Toxins (Basel). 2014; 6:914-933.

[10] Jagannathan J, Sanghvi NK, Crum LA, Yen C-P, Medel R, Dumont AS, Sheehan JP, Steiner L, Jolesz F, Kassell NF (2009). High intensity focused ultrasound surgery (HIFU) of the brain: A historical perspective, with modern applications. Neurosurgery.

[11] Curley CT, Sheybani ND, Bullock TN, Price RJ (2017). Focused Ultrasound Immunotherapy for Central Nervous System Pathologies: Challenges and Opportunities. Theranostics.

[12] Hoogenboom M, Eikelenboom D, den Brok MH, Heerschap A, Fütterer JJ, Adema GJ (2015). Mechanical High-Intensity Focused Ultrasound Destruction of Soft Tissue: Working Mechanisms and Physiologic Effects. Ultrasound Med Biol.

[13] Izadifar Z, Babyn P, Chapman D (2017). Mechanical and Biological Effects of Ultrasound: A Review of Present Knowledge. Ultrasound Med Biol.

[14] Zhou Y-F (2011). High intensity focused ultrasound in clinical tumor ablation. World J Clin Oncol.

[15] Hoogenboom M, Eikelenboom D, den Brok MH, Veltien A, Wassink M, Wesseling P, Dumont E, Futterer JJ, Adema GJ, Heerschap A (2016). *In vivo* MR guided boiling histotripsy in a mouse tumor model evaluated by MRI and histopathology. NMR Biomed.

[16] Jang HJ, Lee J-Y, Lee D-H, Kim W-H, Hwang JH (2010). Current and Future Clinical Applications of High-Intensity Focused Ultrasound (HIFU) for Pancreatic Cancer. Gut Liver.

[17] Timbie KF, Mead BP, Price RJ (2015). Drug and gene delivery across the blood-brain barrier with focused ultrasound. J Control Release.

[18] Treat LH, McDannold NJ, Vykhodtseva NI, Zhang Y, Tam K, Hynynen K (2007). Targeted delivery of doxorubicin to the rat brain at therapeutic levels using MRI-guided focused ultrasound. Int J Cancer.

[19] Kinoshita M, McDannold N, Jolesz FA, Hynynen K (2006). Targeted delivery of antibodies through the blood-brain barrier by MRI-guided focused ultrasound. Biochem Biophys Res Commun.

[20] Kinoshita M, McDannold N, Jolesz FA, Hynynen K (2006). Noninvasive localized delivery of Herceptin to the mouse brain by MRI-guided focused ultrasound-induced blood-brain barrier disruption. Proc Natl Acad Sci U S A.

[21] Etame AB, Diaz RJ, O'Reilly MA, Smith CA, Mainprize TG, Hynynen K, Rutka JT (2012). Enhanced Delivery of Gold Nanoparticles with Therapeutic Potential into the Brain using MRI-Guided Focused Ultrasound. Nanomedicine.

[22] Samiotaki G, Acosta C, Wang S, Konofagou EE (2015). Enhanced delivery and bioactivity of the neurturin neurotrophic factor through focused ultrasound-mediated blood-brain barrier opening *in vivo*. J Cereb Blood Flow Metab.

[23] Wang S, Olumolade OO, Sun T, Samiotaki G, Konofagou EE (2015). Noninvasive, neuron-specific gene therapy can be facilitated by focused ultrasound and recombinant adeno-associated virus. Gene Ther.

[24] Thévenot E, Jordão JF, O'Reilly M a, Markham K, Weng Y-Q, Foust KD, Kaspar BK, Hynynen K, Aubert I (2012). Targeted delivery of self-complementary adeno-associated virus serotype 9 to the brain, using magnetic resonance imaging-guided focused ultrasound. Hum Gene Ther.

[25] Nance E, Timbie KF, Miller GW, Song J, Louttit C, Klibanov AL, Shih T-Y, Swaminathan G, Tamargo RJ, Woodworth GF, Hanes J, Price RJ (2014). Noninvasive delivery of stealth, brain-penetrating nanoparticles across the blood-brain barrier using MRI-guided focused ultrasound. J Control Release.

[26] Mead BP, Mastorakos P, Suk JS, Klibanov AL, Hanes J, Price RJ (2016). Targeted gene transfer to the brain via the delivery of brain-penetrating DNA nanoparticles with focused ultrasound. J Control Release.

[27] Krishna V, Sammartino F, Rezai A (2018). A review of the current therapies, challenges, and future directions of transcranial focused ultrasound technology advances in diagnosis and treatment. JAMA Neurol.

[28] Leinenga G, Götz J (2015). Scanning ultrasound removes amyloid-β and restores memory in an Alzheimer's disease mouse model. Sci Transl Med.

[29] Jordão JF, Thévenot E, Markham-Coultes K, Scarcelli T, Weng YQ, Xhima K, O'Reilly M, Huang Y, McLaurin J, Hynynen K, Aubert I (2013). Amyloid-β plaque reduction, endogenous antibody delivery and glial activation by brain-targeted, transcranial focused ultrasound. Exp Neurol.

[30] Lipsman N, Meng Y, Bethune AJ, Huang Y, Lam B, Masellis M, Herrmann N, Heyn C, Aubert I, Boutet A, Smith GS, Hynynen K, Black SE (2018). Blood-brain barrier opening in Alzheimer's disease using MR-guided focused ultrasound. Nat Commun.

[31] Mainprize T, Lipsman N, Huang Y, Meng Y, Bethune A, Ironside S, Heyn C, Alkins R, Trudeau M, Sahgal A, Perry J, Hynynen K (2019). Blood-Brain Barrier Opening in Primary Brain Tumors with Non-invasive MR-Guided Focused Ultrasound: A Clinical Safety and Feasibility Study. Sci Rep.

[32] Carpentier A, Canney M, Vignot A, Reina V, Beccaria K, Horodyckid C, Karachi C, Leclercq D, Lafon C, Chapelon J-Y, Capelle L, Cornu P, Sanson M, Hoang-Xuan K, Delattre J-Y, Idbaih A (2016). Clinical trial of blood-brain barrier disruption by pulsed ultrasound. Sci Transl Med.

[33] Idbaih A, Canney M, Belin L, Desseaux C, Vignot A, Bouchoux G, Asquier N, Law-Ye B, Leclercq D, Bissery A, De Rycke Y, Trosch C, Capelle L, Sanson M, Hoang-Xuan K, Dehais C, Houillier C, Laigle-Donadey F, Mathon B, André A, Lafon C, Chapelon J, Delattre J, Carpentier A (2019). Safety and Feasibility of Repeated and Transient Blood-Brain Barrier Disruption by Pulsed Ultrasound in Patients with Recurrent Glioblastoma. Clin Cancer Res.

[34] Brenin DR (2011). Focused Ultrasound Ablation for the Treatment of Breast Cancer. Ann Surg Oncol.

[35] Lipsman N, Meng Y, Bethune AJ, Huang Y, Lam B, Masellis M, Herrmann N, Heyn C, Aubert I, Boutet A, Smith GS, Hynynen K, Black SE (2018). Blood-brain barrier opening in Alzheimer's disease using MR-guided focused ultrasound. Nat Commun.

[36] Kovacs ZI, Kim S, Jikaria N, Qureshi F, Milo B, Lewis BK, Bresler M, Burks SR, Frank JA (2017). Disrupting the blood-brain barrier by focused ultrasound induces sterile inflammation. Proc Natl Acad Sci U S A.

[37] Kovacs ZI, Tu T-W, Sundby M, Qureshi F, Lewis BK, Jikaria N, Burks SR, Frank JA (2018). MRI and histological evaluation of pulsed focused ultrasound and microbubbles treatment effects in the brain. Theranostics.

[38] McDannold N, Zhang Y, Vykhodtseva N (2017). The Effects of Oxygen on Ultrasound-Induced Blood-Brain Barrier Disruption in Mice. Ultrasound Med Biol.

[39] McMahon D, Hynynen K (2018). Reply to Kovacs et al.: Concerning acute inflammatory response following focused ultrasound and microbubbles in the brain. Theranostics.

[40] McMahon D, Hynynen K (2017). Acute Inflammatory Response Following Increased Blood-Brain Barrier Permeability Induced by Focused Ultrasound is Dependent on Microbubble Dose. Theranostics.

[41] Kovacs ZI, Burks SR, Frank JA (2018). Focused ultrasound with microbubbles induces sterile inflammatory response proportional to the blood brain barrier opening: Attention to experimental conditions. Theranostics.

[42] McMahon D, Bendayan R, Hynynen K (2017). Acute effects of focused ultrasound-induced increases in blood-brain barrier permeability on rat microvascular transcriptome. Sci Rep.

[43] Silvestrini MT, Ingham ES, Mahakian LM, Kheirolomoom A, Liu Y, Fite BZ, Tam SM, Tucci ST, Watson KD, Wong AW, Monjazeb AM, Hubbard NE, Murphy WJ, Borowsky AD, Ferrara KW (2017). Priming is key to effective incorporation of image-guided thermal ablation into immunotherapy protocols. JCI Insight.

[44] Chavez M, Silvestrini MT, Ingham ES, Fite BZ, Mahakian LM, Tam SM, Ilovitsh A, Monjazeb AM, Murphy WJ, Hubbard NE, Davis RR, Tepper CG, Borowsky AD, Ferrara KW (2018). Distinct immune signatures in directly treated and distant tumors result from TLR adjuvants and focal ablation. Theranostics.

[45] Kheirolomoom A, Silvestrini MT, Ingham ES, Mahakian LM, Tam SM, Tumbale SK, Foiret J, Hubbard NE, Borowsky AD, Ferrara KW (2019). Combining activatable nanodelivery with immunotherapy in a murine breast cancer model. J Control Release.

[46] Yue W, Chen L, Yu L, Zhou B, Yin H, Ren W, Liu C, Guo L, Zhang Y, Sun L, Zhang K, Xu H, Chen Y (2019). Checkpoint blockade and nanosonosensitizer-augmented noninvasive sonodynamic therapy combination reduces tumour growth and metastases in mice. Nat Commun.

[47] Zhang Q, Bao C, Cai X, Jin L, Sun L, Lang Y, Li L (2018). Sonodynamic therapy-assisted immunotherapy: A novel modality for cancer treatment. Cancer Sci.

[48] Schade GR, Wang YN, D'Andrea S, Hwang JH, Liles WC, Khokhlova TD (2019). Boiling Histotripsy Ablation of Renal Cell Carcinoma in the Eker Rat Promotes a Systemic Inflammatory Response. Ultrasound Med Biol.

[49] Bulner S, Prodeus A, Gariepy J, Hynynen K, Goertz DE (2019). Enhancing Checkpoint Inhibitor Therapy with Ultrasound Stimulated Microbubbles. Ultrasound Med Biol.

